# Genetic Background Strongly Modifies the Severity of Symptoms of Hirschsprung Disease, but Not Hearing Loss in Rats Carrying *Ednrb^sl^* Mutations

**DOI:** 10.1371/journal.pone.0024086

**Published:** 2011-09-07

**Authors:** Ruihua Dang, Daisuke Torigoe, Sari Suzuki, Yoshiaki Kikkawa, Kanako Moritoh, Nobuya Sasaki, Takashi Agui

**Affiliations:** 1 Laboratory of Animal Science and Medicine, Department of Disease Control, Graduate School of Veterinary Medicine, Hokkaido University, Hokkaido, Japan; 2 Department of Bioproduction, Tokyo University of Agriculture, Abashiri, Japan; 3 Mammalian Genetics Project, Tokyo Metropolitan Institute of Medical Science, Tokyo Japan; Texas A&M University, United States of America

## Abstract

Hirschsprung disease (HSCR) is thought to result as a consequence of multiple gene interactions that modulate the ability of enteric neural crest cells to populate the developing gut. However, it remains unknown whether the single complete deletion of important HSCR-associated genes is sufficient to result in HSCR disease. In this study, we found that the null mutation of the *Ednrb* gene, thought indispensable for enteric neuron development, is insufficient to result in HSCR disease when bred onto a different genetic background in rats carrying *Ednrb^sl^* mutations. Moreover, we found that this mutation results in serious congenital sensorineural deafness, and these strains may be used as ideal models of Waardenburg Syndrome Type 4 (WS4). Furthermore, we evaluated how the same changed genetic background modifies three features of WS4 syndrome, aganglionosis, hearing loss, and pigment disorder in these congenic strains. We found that the same genetic background markedly changed the aganglionosis, but resulted in only slight changes to hearing loss and pigment disorder. This provided the important evidence, in support of previous studies, that different lineages of neural crest-derived cells migrating along with various pathways are regulated by different signal molecules. This study will help us to better understand complicated diseases such as HSCR and WS4 syndrome.

## Introduction

Hirschsprung disease (HSCR) is a congenital intestinal disease, characterized by the absence of ganglion cells in the distal portion of the intestinal tract. Due to the lack of ganglia, the stool cannot be passed through the colon, and the bowel wall are dilated [1]–[4]. This congenital disorder is observed in about 1/5000 live births, although significant differences in incidence exist between ethnic groups [5]. Several susceptibility genes have been identified for HSCR, namely the *RET* proto-oncogene [6]–[8], endothelin receptor B gene (*EDNRB*) [9]–[16], endothelin-3 gene (*EDN3*) [17], [18], *GDNF* gene [19]–[21], and *SOX10* gene [22], [23], which play important roles in the formation of the enteric nervous system (ENS).

Incomplete penetrance and inter-familial variation are commonly observed in HSCR gene mutations [24]. Moreover, variations in penetrance and severity of aganglionosis between family members carrying equivalent mutations in HSCR genes have also been reported [25]. These lines of evidence suggest that genetic background or multiple gene interactions are important to the development of HSCR disease. This gives rise to the hypothesis that only a single homozygous null mutation of known HSCR-associated genes is insufficient to cause HSCR disease. However, completely homologous deficient mutations in *Ret* [26], *GDNF* [27], *SOX10* [28], *EDNRB* [29], [30], *EDN3* [31] genes have been shown to conformably result in serious aganglionosis phenotype in either human or animal models of HSCR. These results show that such genes play crucial roles in the ontogeny of ENS. In this study, we present our investigation of the impact of genetic background on the penetrance and severity of aganglionosis in rat strains with the same null mutation of the *Ednrb* gene.

The *Ednrb* gene encodes a G-protein-coupled seven-transmembrane receptor that interacts with a family of ligands known as endothelins that are known to be associated with HSCR disease [29]. Spotting lethal (*sl*) is a spontaneous null mutation that has a 301 bp deletion starting from nucleotide 229 of exon 1, and spanning the entire 3′ end of exon 1 and the first 44 bp of intron 1 in the rat *Ednrb* gene [32]. This mutation abrogates the authentic splice donor site and results in the absence of a functional receptor protein [32], [33]. Homozygous *Ednrb^sl^*-mutated rats show a serious aganglionosis phenotype. In this study, we produced an AGH-*Ednrb^sl^* inbred strain by the brother-sister mating of heterozygous rats for 21 generations, and then evaluated the impact of genetic background on aganglionosis symptoms by constructing congenic strains carrying *Ednrb^sl^* mutations. Results showed that the *Ednrb* gene is not indispensable to ENS development.

In addition, we also investigated tone burst-evoked auditory brainstem response (ABR) in wild type, heterozygous, and homozygous *sl* rats to determine hearing levels. Interestingly, we found this mutation results in seriously congenital sensorineural deafness. Homozygous AGH-*Ednrb^sl/sl^* rats also demonstrate a loss of skin pigment, thereby reproducing the characteristic features of Waardenburg–Shah Syndrome 4 (WS4) in humans, including aganglionosis of the colon, depigmented patches of skin, and sensorineural hearing loss, and may serve as an ideal model of WS4 [34]. In populations of human WS4 patients, high inter- and intrafamilial phenotype variability was also observed [34]. However, whether these phenotypic discrepancies result from the mutation involved or from the genetic background remains to be determined. Because of the small sample number and heterogeneous genetic background in human patients, we cannot precisely evaluate the relationship between the mutation or genetic background and disease phenotype. The availability of this WS4 model offers hope that the effects of genetic background on the three features of WS4 syndrome may be precisely evaluated and may also lead to a more precise understanding of WS4 pathophysiology. In this study, we used three strains of *Ednrb^sl/sl^* rats to focus on how and to what extent genetic background impacts the three features of WS4 syndrome as well as the relationships between the three features.

Sex bias is an important characteristic of HSCR, the incidence of which is 4 times greater in male infants than in females [4]. We haven't seen such a large sex bias to date in animal models, and the mechanism underlining the sex bias remains to be determined. In this study, we also explored whether genetic background change can lead to sex bias. We found a trend that higher penetrance existed in males than in females in strains with short segment aganglionosis.

## Results

### Genetic backgrounds strongly affect the penetrance and severity of aganglionosis in *Ednrb^sl/sl^* rats

Homozygous AGH-*Ednrb^sl/sl^* rats show an aganglionosis phenotype. A previous report shows that the *sl* mutation exists as a 301 bp deletion in exon 1 through intron 1 in the *Ednrb* gene and is responsible for this phenotype [32]. We introgressed this mutation into LEH and F344 strains to produce two congenic strains: LEH-*Ednrb^sl^* and F344-*Ednrb^sl^*. The *Ednrb* gene locates on chromosome 15 in the rat, so we used 33 microsatellite markers (Table S1) to examine the congenic extent to which the genetic background was changed. The results showed that genetic background was replaced into the 3 cM region around the *Ednrb* gene in both the LEH and F344 congenic strains (Fig. S1). In both congenic strains, part or all homozygous rats with *sl* mutations showed symptoms of aganglionosis. This provided further evidence to confirm that the *Ednrb^sl^* mutation is the dominant cause of aganglionosis.

**Figure 1:**
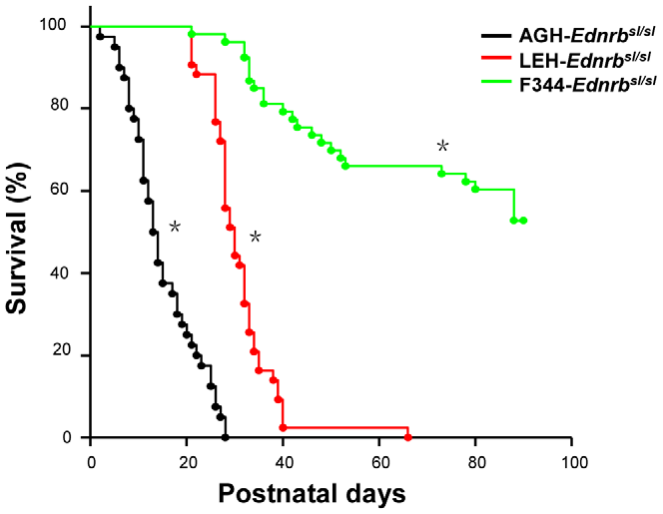
Comparison of survival curves in rats from the three strains until postnatal 90 days. Logrank test shows significant difference in survival curve among the three strains(p<0.01, *). AGH-*Ednrb^sl/sl^*, n = 40; LEH-*Ednrb^sl/sl^*, n = 42; F344-*Ednrb^sl/sl^*, n = 53.

**Figure 2:**
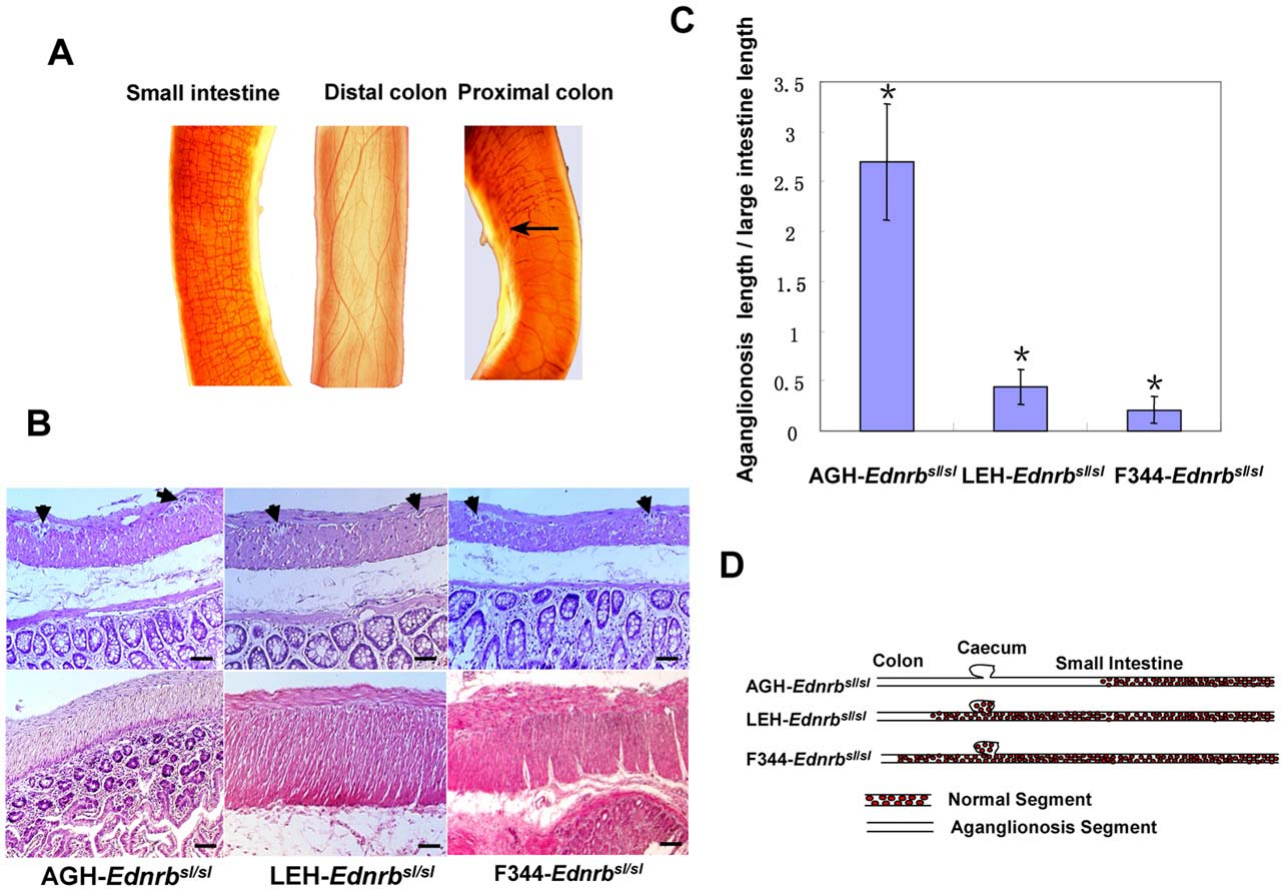
AChE whole-mount staining and HE staining of the intestine. A. AChE whole-mount staining to visualize the ganglia architecture in the ENS of LEH-*Ednrb^sl/sl^* rats. Ganglia can be seen in the small intestine, but not in the distal colon. The boundary of the normal and aganglionosis intestine can be discerned in the proximal colon. The arrow indicates the boundary. B. HE pathological tissue examination confirmed that no ganglions were present in the aganglionosis segment. Upper panels were taken from normal intestine tissue of the three strains. Lower panels were taken from the aganglionosis segment. Arrows indicate ganglias. Scale bar: 10 µm. C. A comparison of the extent of aganglionosis in rats from the three strains. The ratio of the extent of aganglionosis in AGH (n = 33) was significantly higher than those in LEH (n = 34) and F344 (n = 35) rats (p<0.01, *). Data are shown as mean ± SD. D. Diagram of the extent of aganglionosis in rats from the three strains.

By producing these congenic lines, the impact of genetic background on the features of aganglionosis can be precisely evaluated. Three lines of homozygous rats with the same *Ednrb* mutation exhibit distinct degrees of severity of the megacolon. In AGH-*Ednrb^sl/sl^* rats, only 20% of infants survived until weaning; whereas in LEH-*Ednrb^sl/sl^* and F344-*Ednrb^sl/sl^* rats, 100% of infants survived to weaning, although 90% of LEH-*Ednrb^sl/sl^* pups died before postnatal 40 day (Fig. 1). There were significant differences in survival curve between the three lines (P<0.01). Interestingly, almost 60% of F344-*Ednrb^sl/sl^* pups did not show any symptoms of aganglionosis (Table 1), appearing healthy and normally fertile and showing normal body weight gain, unlike AGH-*Ednrb^sl/sl^* and LEH-*Ednrb^sl/sl^* rats, which showed decreases in weight due to megacolon. To confirm that the differences in survival and penetrance between AGH-*Ednrb^sl/sl^*, LEH-*Ednrb^sl/sl^* and F344-*Ednrb^sl/sl^* rats corresponded to the severity of intestinal aganglionosis phenotype, we evaluated the extent of aganglionosis in postnatal day 14 pups in these strains by whole-mount acetylcholinesterase (AChE) staining [35]. AChE staining can rapidly detect cholinergic neurons and visualize ganglia architecture in the ENS (Fig. 2A), and it makes it possible to directly observe the three-dimensional organization of the intestinal tissue. To further test its precision, we undertook pathological tissue analysis and confirmed the absence of ganglions in the aganglionosis intestine in the three strains of homologous mutant rats (Fig. 2B). The extent of aganglionosis in the three strains of AGH-*Ednrb^sl/sl^* (n = 33), LEH-*Ednrb^sl/sl^* (n = 34) and F344-*Ednrb^sl/sl^* (n = 35) rats was calculated as a ratio of length of the aganglionosis intestine to the length of the entire large intestine. A hundred percent of AGH-*Ednrb^sl/sl^* rats exhibited aganglionosis reaching beyond the caecum, whereas none of the LEH-*Ednrb^sl/sl^* or F344-*Ednrb^sl/sl^* rats showed aganglionosis above the caecum (Fig. 2C). In LEH-*Ednrb^sl/sl^* rats, aganglionosis was mostly limited to the middle colon. In F344-*Ednrb^sl/sl^* rats, aganglionosis appeared in a very short segment near anus or not at all (Fig. 2C). The differences in the extent of aganglionosis between the three lines were also statistically significant (P<0.01). To clearly exhibit the extent of the aganglionosis, diagrams of the representative features of innervation in the three strains are shown in Fig. 2D. Among three strains of homologous rats, the AGH-*Ednrb^sl/sl^* rats showed the most serious aganglionosis, followed by the LEH-*Ednrb^sl/sl^* and F344-*Ednrb^sl/sl^* rats, which corresponded to the order of their average survival time. Thus, we concluded that variations in penetrance and survival period among the three strains of *Ednrb^sl/sl^* rats was attributable to distinct differences in the severity of aganglionosis, and modifier genes in the genetic backgrounds of these strains significantly modulated the severity of the aganglionosis phenotype.

**Table 1:** Incidence of aganglionosis in *Ednrb^sl^* ^/*sl*^ rats of three strains.

Phenotype	AGH-*Ednrb^sl/sl^*		LEH-*Ednrb^sl/sl^*		F344-*Ednrb^sl/sl^*	
	Males	Females	Males	Females	Males	Females
Normal (n)	0	0	0	0	15	13
Aganglionosis (n)	17	15	12	15	17	8
Incidence (%)	100%	100%	100%	100%	53%	38%

Total numbers of rats: AGH-*Ednrb^sl/sl^*, n = 32; LEH-*Ednrb^sl/sl^*, n = 27; F344-*Ednrb^sl^* ^/*sl*^, n = 53.

### Homozygous *sl* mutations in the *Ednrb* gene result in serious congenital sensorineural deafness, whereas genetic background only slightly affects the severity of sensorineural deafness in *Ednrb^sl/sl^* rats

**Figure 3:**
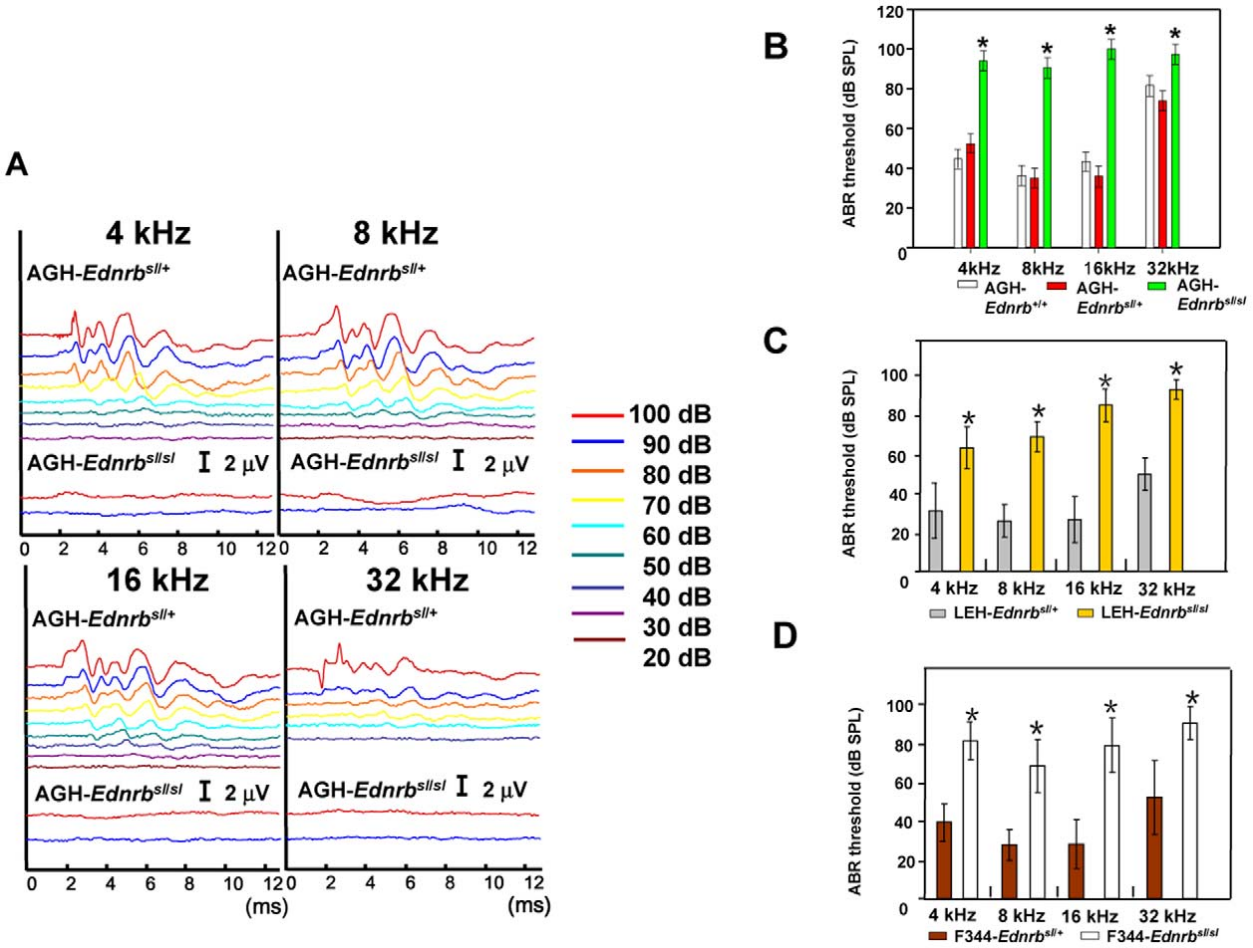
Congenital deafness in AGH-*Ednrb^sl/sl^* rats. A. ABR waveforms of 18-day-old littermate AGH-*Ednrb^sl/+^* (the upper) and AGH-*Ednrb^sl/sl^* (the lower) rats at 20–100 dB SPL at 4–32 kHz is presented on the same scale (2.0 mV) for clarity. B. Hearing levels (mean ± SD) in 18-day-old AGH-*Ednrb^sl/sl^* rats (green squares, n = 4), littermate heterozygous rats (red squares, n = 8) and WT rats (white squares, n = 4) were measured by ABR. The hearing levels of AGH-*Ednrb^sl/sl^* rats were significantly lower than those of other littermates (P<0.01, *). C. Hearing levels (mean ± SD) in 18-day-old LEH-*Ednrb^sl/sl^* rats (yellow squares, n = 6) were significantly lower than those of littermate heterozygous rats (gray squares, n = 6). D. Hearing levels (mean ± SD) in 18-day-old F344-*Ednrb^sl/sl^* rats (white squares, n = 8) were significantly lower than those of littermate heterozygous rats (red squares, n = 18).

**Figure 4:**
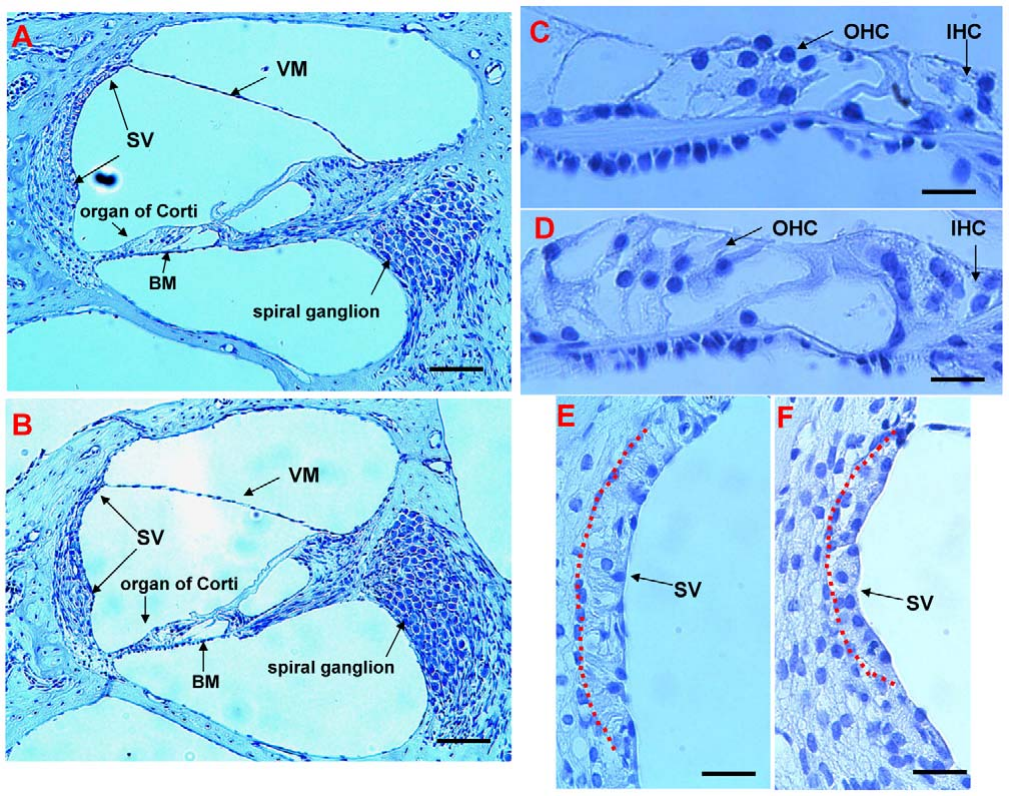
HE-staining of a cross-section of the cochlea from the temporal bones. HE staining of a cross section of the cochlea from the temporal bones in 18-day-old AGH-*Ednrb^sl/+^* (A) and AGH-*Ednrb^sl/sl^* (B) rats. No OHC or IHC abnormalities were found in AGH-*Ednrb^sl/sl^* rats (D) compared with those in AGH-*Ednrb^sl/+^* rats (C). An enlarged stria vascularis was observed in AGH-*Ednrb^sl/+^* rats (E), which was thicker than that in AGH-*Ednrb^sl/sl^* rats (F). These results were confirmed by using three different rats. Scale bars: (A, B) 100 µm; (C, D, E, F) 25 µm. Stria vascularis (SV); Vestibular membrane (VM); Basilar membrane (BM); Outer hair cells (OHC); Inner hair cells (IHC).

*Ednrb* and *Sox10* mutations are frequently associated with WS4. Mice with *Ednrb* mutation are characterized by sensorineural deafness and aganglionosis [36]. Recently, it was reported that among mice with different *Ret* mutations, only *c-Ret*-KI^Y1062F/Y1062F^ mice have sensorineural deafness, suggesting that a locus mutation effect may exist [37]. To examine whether this *Ednrb* mutation in rats affects hearing levels, we investigated tone burst-evoked auditory brainstem response (ABR) in AGH rats of three genotypes: *Ednrb^+/+^*, *Ednrb^+/sl^* and *Ednrb^sl/sl^*. Thresholds for sound at 4–32 kHz in 18-day-old AGH-*Ednrb^sl/sl^* rats [90- to 100-dB sound pressure level (SPL)] were much higher than those in heterozygous or WT littermates (40- to 80-dB SPL) (P<0.01) (Fig. 3B). The latencies of all ABR waves in the *Ednrb^sl/sl^* rats were also very prolonged (Fig. 3A). These results suggested that *Ednrb^sl/sl^* rats suffered from severe congenital deafness. When this mutation was introgressed into different genetic backgrounds, it still led to hearing loss (Fig. 3C, 3D). Despite the survivorship curve described earlier in the paper, a few unanticipated homozygous survivors in LEH strain were discovered that were used to test hearing. Hearing levels in 10-week-old adult LEH-*Ednrb^sl/sl^* and F344-*Ednrb^sl/sl^* rats were also lower than those in heterozygous rats (Fig. S2). To find the pathological cause, we performed a histological examination of the cochleae of AGH-*Ednrb^sl/sl^* rats at postnatal 18 days. Interestingly, we found only that the stria vascularis was thinner in AGH-*Ednrb^sl/sl^* rats compared to heterozygous rats, with none of the other abnormalities, such as endolymphatic collapse, Reissner membrane shift, deteriorated Corti, or hair cell loss, found in WS4 mice (Fig. 4) [36].

We next investigated to what extent hearing loss can be modified by genetic background in *Ednrb^sl/sl^* rats. The sound thresholds at 8–32 kHz in 18-day-old *Ednrb^sl/sl^* rats appeared to correspond to changes in aganglionosis severity among the three strains. Namely, AGH-*Ednrb^sl/sl^* rat showed the most serious hearing loss, followed by LEH-*Ednrb^sl/sl^* rat and F344-*Ednrb^sl/sl^* rat (Fig. S3), although the differences were not statistically significant. This result suggested that, unlike aganglionosis, the *EDNRB*-mutation-related hearing loss was not readily affected by genetic background.

### Genetic backgrounds also affect the severity of pigmentation abnormalities, but no correlation exists between aganglionosis and pigmentation loss in *Ednrb^sl/sl^* rats

**Figure 5:**
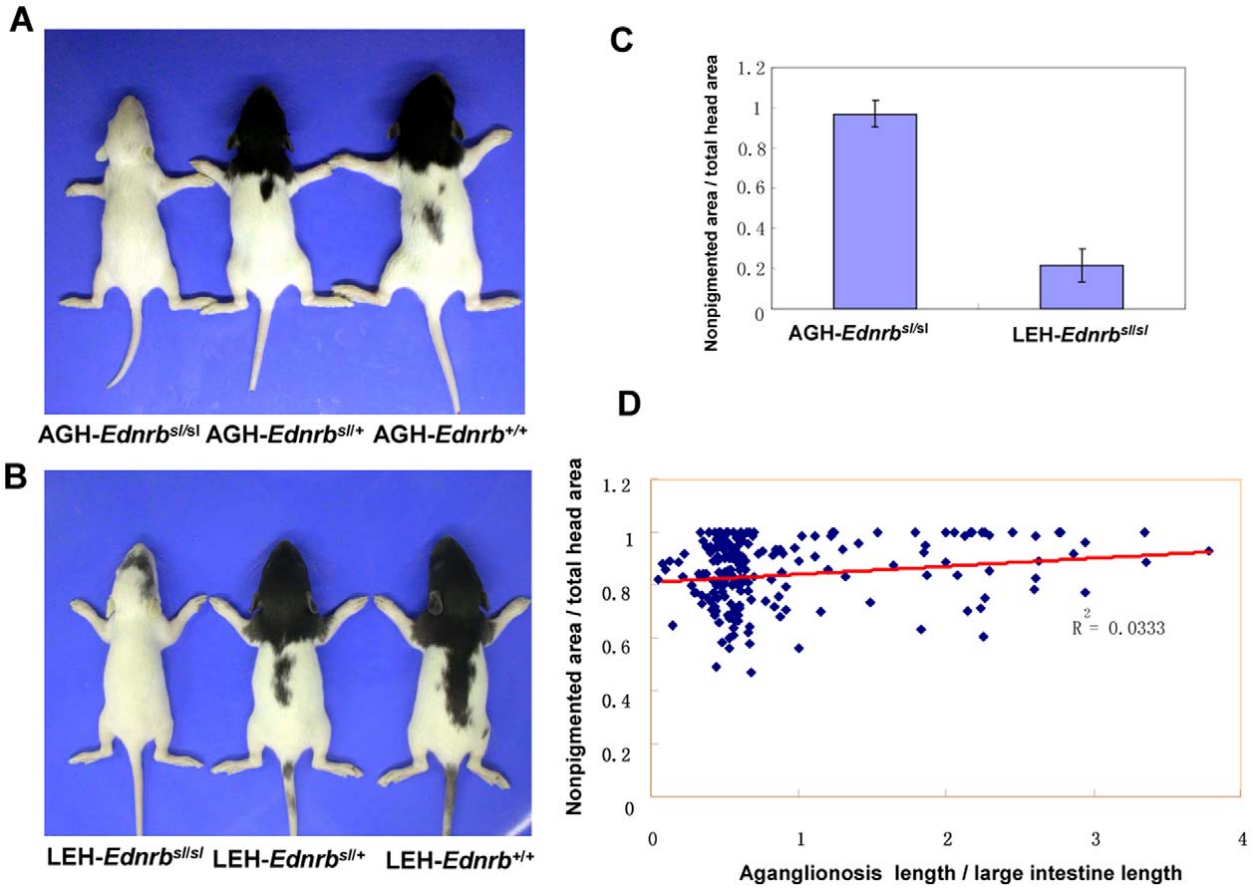
Correlation analysis between pigmentation abnormality and extent of aganglionosis. AGH-*Ednrb^sl^* (A) and LEH-*Ednrb^sl^* (B) from the three genotypes. The ratio of nonpigmented area/total head area in AGH- *Ednrb^sl/sl^* rats is distinctly higher than that in LEH- *Ednrb^sl/sl^* rats (C). Data are shown as mean ± SD. Correlation analysis between pigmentation abnormality and the extent of aganglionosis shows very low correlation between the two features (D).

This mutation in the *Ednrb* gene also resulted in pigmentation disturbance in rats. Wildtype and heterozygous AGH-*Ednrb^sl^* rats had pigmented heads, backs, and tails. However, homozygous rats with a *sl* mutation had almost no pigmentation on their heads apart from black eyes (Fig. 5A). When this mutation was bred onto the LEH background, LEH-*Ednrb^sl/sl^* rats exhibited a large pigmented spot on the head (Fig. 5B). This demonstrated that the pigmentation disturbance could also be rescued to some extent. We hypothesized that the same modifier genes in the LEH background rescued both aganglionosis and pigmentation disturbance, both of which arise from defects in the migration or proliferation of neural crest-derived cells [38]. To examine this hypothesis, we produced an F_2_ population (AGH-*Ednrb^sl^*×LEH-*Ednrb^sl^*) to analyze whether there was a correlation between the two phenotypes. The extent of pigment loss was calculated as the ratio of unpigmented area to total area of the head, which was distinctly higher in LEH-*Ednrb^sl/sl^* than that in AGH- *Ednrb^sl/sl^* rats (Fig. 5C). The results showed that there was no significant correlation between the aganglionosis and pigmentation abnormality in *Ednrb^sl/sl^* rats in the F_2_ population (Fig. 5D). This indicated that the modifier genes of the two phenotypes in the LEH background were different.

### Sex bias in the penetrance of aganglionosis exists in populations with short segment aganglionosis

Sex bias is an important feature of HSCR disease in humans. Males are almost four times more often affected than are females, a difference most prominent in short segment HSCR (S-HSCR, 80% case) [39]. In our study, we attempted to identify this phenomenon in an animal model, and analyzed the relation between genetic background and the occurrence of sex bias. Sex bias might be the result of an unbalanced embryo that is lethal in one gender. Therefore, we genotyped a large amount of pups from the three lines. All genotypes were present in the expected Mendelian ratio, even in mixed genetic backgrounds of F_1_ and F_2_ populations (AGH-*Ednrb^sl^*×F344-*Ednrb^sl^* or AGH-*Ednrb^sl^*×LEH-*Ednrb^sl^*) (Table S2). This demonstrated that there was no lethal unbalanced embryo in these populations. Next, we investigated whether there was a penetrance bias between males and females in each strain. AGH-*Ednrb^sl/sl^* and LEH-*Ednrb^sl/sl^* rats showed 100% penetrance in both males and females. Interestingly, we observed a sex bias with regard to penetrance in the F344-*Ednrb^sl/sl^* population, which demonstrates the mildest degree of aganglionosis among these strains. The penetrance in male homozygous rats was higher than that in females (Table 1), suggesting that the sex bias in penetrance was more likely to occur in pups that showed short segment aganglionosis. To further confirm this hypothesis, we continued to observe the progenies of the intercross population between the normal male and female F344-*Ednrb^sl/sl^* rats. Consistent with this hypothesis, we observed a nearly two times higher penetrance in males than females in this population (data not shown).

**Figure 6:**
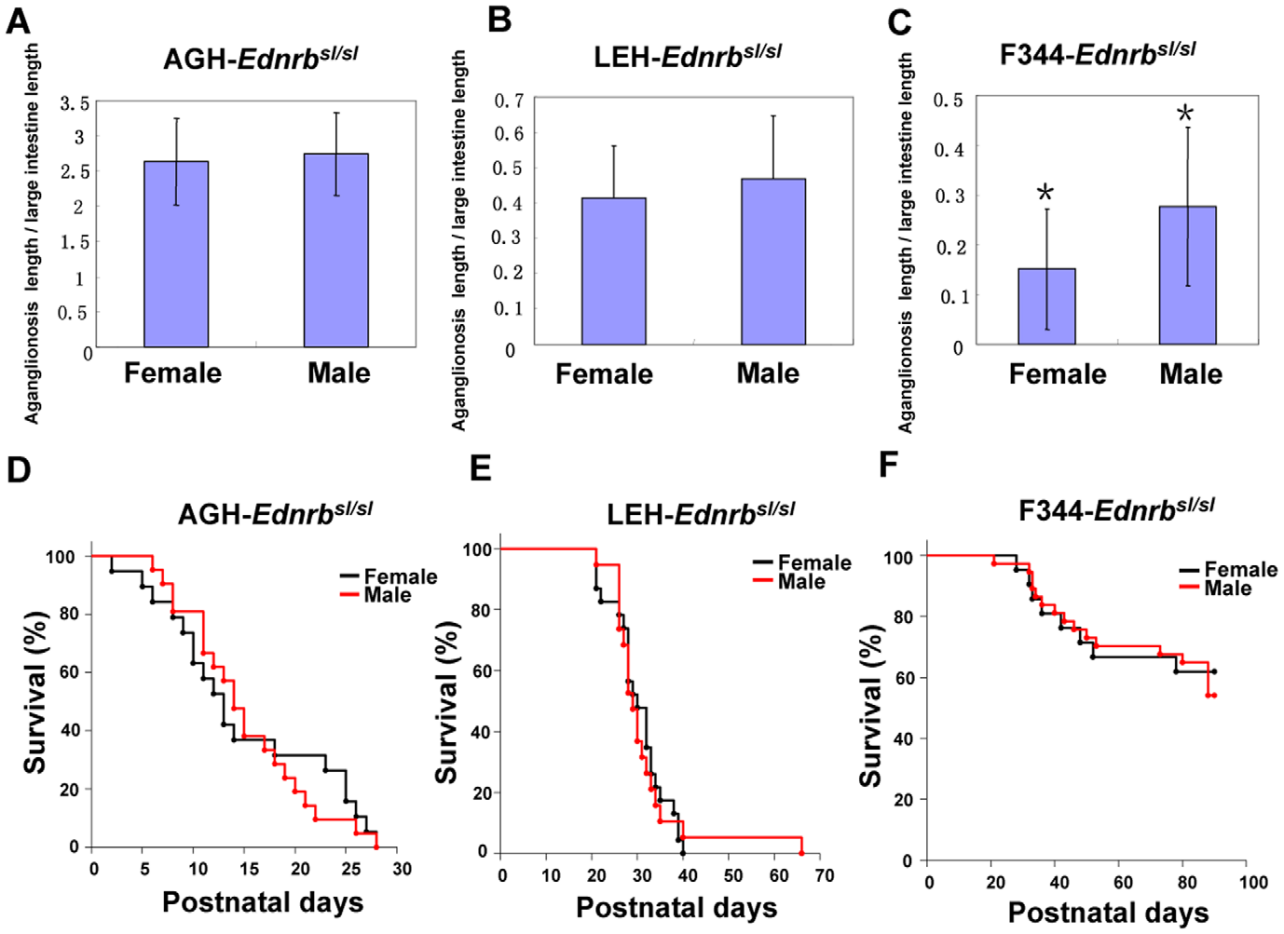
Comparison of the extent of aganglionosis and survival time between genders in homologous rats from the three strains. The extent of aganglionosis between genders in AGH- *Ednrb^sl/sl^* (A), LEH- *Ednrb^sl/sl^* (B), and F344-*Ednrb^sl/sl^* (C) rats was compared. Only in F344-*Ednrb^sl/sl^* rats was the aganglionosis ratio in male rats (n = 17) significantly higher than that in female rats (n = 18) (p<0.05,*). Data are shown as mean ± SD. Survival curves between genders in AGH- *Ednrb^sl/sl^* (males, n = 21; females, n = 19) (D), LEH-*Ednrb^sl/sl^* (males, n = 19; females, n = 23) (E), and F344-*Ednrb^sl/sl^* (males, n = 32; females, n = 21) (F) rats until postnatal 90 days was compared. No significant differences were found.

Moreover, as it is known that survival period is associated with the extent of intestinal aganglionosis, it is probable that there was a gender-based difference in the extent of intestinal aganglionosis in F344-*Ednrb^sl/sl^* rats. To test this hypothesis, we compared the extent of aganglionosis between male and female homozygous pups from the three strains. We found no significant differences in the extent of aganglionosis between male and female pups in the AGH-*Ednrb^sl/sl^* and LEH-*Ednrb^sl/sl^* rats (Fig. 6A, B). However, in the F344-*Ednrb^sl/sl^* rats, the extent of aganglionosis in males was significantly higher (p<0.05) than that in females (Fig. 6C). We also compared the survival curves until postnatal 90 days between male and female homozygous rats from the three strains; however, no significant differences were found (Fig. 6D, E, F), which showed that the gender modifier effect only occurred in pups with mild aganglionosis decreasing to a certain level.

## Discussion

Normal ENS formation includes complicated biological processes, which starts with the migration of neural crest cells to populate the gastrointestinal tract, followed by expansion of an initially small cell population, and result in the creation of multiple cell lineages [2], [40], [41]. Neural crest cells from both the vagal and sacral levels of the neural tube colonize the gut, where they proliferate and differentiate into neurons and glia [41], [42]. The multi-step complex nature of ENS ontogeny makes it highly susceptible to alterations in gene function or expression. In human populations, the fact that HSCR penetrance in many families is at least in part determined by the presence of more than one mutant allele suggests that HSCR is the consequence of multiple gene interactions that modulate the ability of enteric NC cells to populate the developing gut. In mice, heterozygosity for two known mutant HSCR genes, such as the *Ret* and *Ednrb* [43], *Sox^Dom/+^* and *EDNRB*, or *Sox^Dom^* ^/+^ and *EDN3* genes [44], also leads to intestinal aganglionosis, whereas mice heterozygous for either allele alone have no intestinal or shorter segments of aganglionosis. Thus, the synergistic effects of multiple hypomorphic mutation in HSCR-associated genes can influence disease penetrance and expressivity. It is thought that a single mutation at an individual locus may be neither necessary nor sufficient to cause clinical disease. Our study provided new evidence in support of this opinion. *EDNRB* is required during a restricted period of neural crest development between embryonic days 10 and 12.5 [45]. *Ednrb*-knockout mice showed serious megacolon and died at 2–4 weeks of age [30]. Similar phenotypes were observed in lethal spotting (*ls*) mice that harbor null mutations in the *Edn3* gene, which encodes a ligand for *EDNRB* [31]. Mutations in the *Edn3* and *Ednrb* genes also result in aganglionosis in other mammals [46], [47]. Furthermore, targeting of the *Ednrb* gene to the neural crest also resulted in megacolon in mice [48]. All these results suggest that the *Ednrb* gene plays a critical role in the development of neurons and glia in the ENS. Surprisingly, the complete deletion of the *Ednrb* gene in about 60% of F344-*Ednrb^sl/sl^* rats did not result in HSCR disease. This demonstrated that *EDNRB* was not indispensable for ENS development and led to a significant change in our thinking regarding the relationship between the *Ednrb* gene and HSCR disease. *Ret*-knockout mice died soon after birth due to renal agenesis and the lack of enteric neurons throughout the digestive tract [26]. Complete loss of *Sox10* led to a total absence of enteric ganglia in homozygotes [28]. *Gdnf*-null mice showed a complete lacking of the enteric nervous system, ureters, and kidneys [27]. Our study suggested if they are bred onto different backgrounds it is also possible that these deficient mutations do not cause aganglionosis symptoms. The reason for the partly rescued aganglionosis phenotype in F344-*Ednrb^sl/sl^* rats in this study remains unclear; however, modifier genes might be differentially expressed in this strain to modulate or compensate for the effect of the *Ednrb* deficiency. In addition, differences in the patterns of inheritance between animal models and humans should be noted. Heterozygous rats with a null allele of the *Ednrb* receptor or ligand remain asymptomatic for hypopigmentation and colonic aganglionosis. In contrast, humans harboring one heterozygous allele demonstrate short segment aganglionosis in the presence of hypopigmentation. In any case, however, the current results will greatly enrich our understanding of HSCR disease.

Pluripotent neural-crest cells migrate from the neural tube throughout the embryo along several pathways and give rise to different cell types, including glia and neurons in the peripheral nervous system, some of the craniofacial skeletal tissue, and melanocytes in the skin and inner ear [38]. The vast majority of cells in the mammalian inner ear, including the sensory epithelia and neurons, are derived from the otic placode [49]. Intermediate cells in the stria vascularis of the cochlea are melanocytes arising from neural crest cells, on plasma membrane of which a K^+^ channel family, Kir4.1, play a critical role in the generation of the K^+^ gradient between the plasma membrane and the endolymph fluid, which is essential for the transduction of sound by hair cells [50]. One gene mutation associated with neural-crest development commonly causes multiple tissue defects. In humans, *EDNRB* mutations are frequently associated with sensorineural deafness in addition to aganglionosis, indicating that the *Ednrb* gene plays a role in the migration and proliferation of neural crest-derived cochlea melanocytes. In this study, we confirmed that the mutation of the *Ednrb* gene in rats also resulted in serious sensorineural deafness, thereby demonstrating that the function of the *Ednrb* gene is conservative for cochlea melanocytes. In addition, we found only slight tissue differences in the cochlea between normal and deaf homozygous rats, which differs from the findings of distinct abnormal inner ear structures in WS4 mice. The absence of melanocytes in the cochlea of mutant mice appears to disrupt ion homeostasis, leading to structural collapse and subsequent loss of the sensory epithelium [36]. In mutant rats, it is possible that, due to the disruption of ion homeostasis resulting from the loss of melanocytes in the stria vascularis, the transduction of sound by hair cells is impeded. In future, we will continue to explore this mechanism in greater detail. Homozygous rats with an *EDNRB* mutation in different genetic backgrounds show approximately equal hearing loss, which suggested that hearing level is not readily influenced by genetic background and that the neural crest-derived cochlea melanocytes are sensitive to *EDNRB* mutation. However, another possibility that cannot be denied is that there is no strong modifier genes for hearing phenotype in the genetic backgrounds of the two congenic strains used in this study. In humans, mutation of the *Ednrb* gene is not associated beyond doubt with congenital deafness [25]. This demonstrated a difference in inheritance between humans and the animal models.

The *Ednrb* gene is also implicated in the development of another neural crest derived-cell lineage: melanocytes [45]. Homozygous AGH-*Ednrb^sl/sl^* and LEH-*Ednrb^sl/sl^* rats both show pigmentation loss in their heads, but to different extents that correspond to aganglionosis severity. Melanocytes and enteric neurons all arise from the neural crest, so it is possible that the same modifier genes affect the two neural crest-derived lineages. However, results of the correlation analysis between the two phenotypes in the F_2_ population (AGH-*Ednrb^sl^*×F344-*Ednrb^sl^*) did not support this hypothesis. These data provided new evidence in support of previous results regarding development biology that suggested distinct signaling molecules control the migration of neural crest cells in the different migration pathways [38].

Sex bias is an interesting phenomenon observed in HSCR patients, where the disease is about 4 times more commonly in males than in females [4]. The molecular basis for this gender-dependent disease penetrance has not, however, been defined. It is possible that modifier genes in the sex chromosome lead to this result. However, until now, no related genes were identified in humans. Recent reports have suggested that gender bias in the extent of distal intestinal aganglionosis also occurs in mice [43]. Our studies demonstrated an interesting result: in the strain showing short segment aganglionosis, there was a gender bias in penetrance that corresponded to the extent of aganglionosis, whereas in the strains that showing longer segment aganglionosis with the same mutation, no gender bias in penetrance or extent of aganglionosis was observed. In Ret^9/−^ mice, which showed short segment aganglionosis, there was also a tendency for males to be affected more frequently than females [51]. These data suggested that the influence of gender on penetrance, mostly in rat or mouse strains with short segment aganglionosis, is irrelevant to mutation genes, which was similar to an observation in humans that sex bias mostly occurs in short segment cases [39]. Taken collectively, F344-*Ednrb^sl/sl^* rats represented typical features of HSCR (incomplete penetrance and sex bias), and for this reason may serve as a valuable model for HSCR disease.

One mutation of the *Ednrb* gene caused three symptoms: aganglionosis, pigmented disorder and hearing loss. When bred onto different genetic backgrounds, these features were differently modified. AGH-*Ednrb^sl/sl^* and LEH-*Ednrb^sl/sl^* rats reproduced features of WS4 in humans, characterized by intestinal aganglionosis, hypopigmentation and hearing loss. Normal F344-*Ednrb^sl/sl^* rats only exhibited hearing loss (pigmentation defects could not be assessed as they were albino), constituting a rat model of WS2, which does not demonstrate aganglionosis. The same genetic background exerted a largely different effect on the three symptoms resulting from the same mutation. This fully explained the reason for the complexity of WS4 Syndrome in humans, namely that different modifier genes in the genetic background played crucial roles in occurrence of these symptoms.

In conclusion, this study has shown that genetic background plays a key role in determining the final impact of *Ednrb* mutation. The null mutation of the *Ednrb* gene commonly results in three features of WS4 syndrome. However, these features are significantly and variously modulated by genetic background. Studies such as this that explore both disease-causing mutation and the contribution of genetic background are vital to increasing our understanding of complicated diseases such as HSCR and WS4 syndrome.

## Materials and Methods

### Animals

An original closed colony of aganglionosis rats and a closed colony of Long-Evans rats, both of which carry the *Ednrb^sl^* mutation, were provided by Dr. Ozaki, National Institute for Physiological Sciences, Okazaki, Japan. F344 rats were purchased from SLC (Hamamatsu, Japan). Heterozygous female and male rats were brother-sister-mated over 21 generations to produce inbred strains carrying the *Ednrb^sl^* mutation, which were thereafter named AGH/Hkv (aganglionosis Hokkaido)-*Ednrb^sl^* and LEH/Hkv (Long-Evans Hokkaido)-*Ednrb^sl^*. An F344-*Ednrb^sl^* congenic line was established by backcrossing original aganglionosis rat carrying the *Ednrb^sl^* mutation to the F344 inbred strain over 10 generations and then maintained by brother-sister-mating. Animals were maintained in specific pathogen-free conditions with feeding and drinking allowed *ad libitum*. A humane end point was applied when the *sl* homozygous rats with severe megacolon became moribund. All research and experimental protocols were conducted according to the Guidelines for the Care and Use of Laboratory Animals of the Graduate School of Veterinary Medicine of Hokkaido University and were approved by the Animal Care and Use Committee of Hokkaido University (Approval ID: No. 110226).

### Genotyping

DNA was extracted from tail clips using standard methods. Animals were genotyped for *Ednrb^sl^* mutation using primers (F-CCTCCTGGACTAGAGGTTCC and R-ACGACTTAGAAAGCTACACT) that flank the site of the 301-base deletion. PCR products were electrophoresed in 2% agarose gels to distinguish the wild (511 bp) and mutant (210 bp) alleles (Fig. S4).

### Histological analysis

The guts from pups at postnatal day 14 were dissected as a single piece from the proximal esophagus to the distal colon. Mesenteric attachments and the pancreas were removed, and the guts were then processed for acetycholinesterase whole-mount staining (AChE) using routine protocols to visualize enteric ganglia [35]. The extent of the gut regions affected by aganglionosis was determined by microscopic examination. The entire length of the gut, as well as any aganglionic regions, was measured. The length of the aganglionic segment was divided by the whole colon length to yield an aganglionosis ratio. For HE staining, gut tissues were dissected and fixed in 10% formalin in a PBS solution. Embedded tissues were cut into 5-µm sections and stained with hematoxylin and eosin by the standard procedure. Sections were examined under bright-field microscopy. For pathological analysis of the cochlea, rats at postnatal 18 day were decapitated under anesthesia, and the temporal bones were removed, fixed with 4% paraformaldehyde, and then decalcified for 10 days with 5% EDTA/PBS. The paraffin-embedded tissues were cut and stained with hematoxylin.

### Measurement of Hearing

The ABR testing of hearing threshold in rats was performed with a tone pip stimulus (4, 8, 16 and 32 kHz), using an evolved potential recording system (TDT/ADI). Both right and left ears from rats of each strain were used for the ABR measurement. Rats were first anesthetized with an intraperitoneal injection of pentobarbital (60–70 mg/kg), and ABRs were recorded with stainless steel needle electrodes inserted subcutaneously into the vertex (active), one side of the retroauricular region (inactive) and the opposite thigh (ground). For each frequency, a stimulus sound pressure level (in decibels; dB SPL) as a tone pip, consisting of 0.1 ms slope, 1-ms duration and a 50-ms repeat interval, was delivered in a free field. A sound source (speaker) was inserted into the external acoustic meatus of both ears of each mouse. The maximum SPL presented for all stimuli was 100 dB. ABR thresholds were obtained for each stimulus by reducing the SPL first at 10 dB steps and finally at 5 dB steps up and down to identify the lowest level at which an ABR pattern could be recognized. Data are presented as mean ± SD.

### Measurement of unpigmented coat ratio

Photographs of the dorsal side of the rats were taken with a COOLPIX 4500 digital camera (Nikon, Tokyo, Japan). To control for variations in size among the animals, the ratio of unpigmented area (unpigmented area/total head surface area) was calculated by image processing using Photoshop Elements 4.0 (Adobe Systems, California, USA).

### Statistical analyses

Kaplan-Meier analysis was performed to produce survival curves. For comparison of survival curves, the logrank test was used to determine significant differences between groups. One-way ANOVA was used to compare the extent of aganglionosis among *Ednrb^sl/sl^* rats of the three strains and hearing levels among AGH- *Ednrb^sl/sl^* rats of the three genotypes. Other statistical analyses were performed using t-test to compare the mean values for data sets.

## Supporting Information

Figure S1 **Diagram of LEH-** ***Ednrb^sl^*** **and F344-** ***Ednrb^sl^*** **congenic extent.** Thirty-three microsatellite markers located on chromosome 15 in the rat were used to examine the congenic extent of LEH-*Ednrb^sl^* and F344-*Ednrb^sl^* rats. Genetic background in both strains of rat was replaced by the 3 cM region around the *Ednrb* gene.
(TIF)

Figure S2 Hearing levels (mean ± SD) in 10-week-old LEH-*Ednrb^sl/sl^* rats (gray squares, n = 2) and littermate heterozygous rats (white squares, n = 4), as well as in F344-*Ednrb^sl/sl^* rats (yellow squares, n = 4) and littermate heterozygous rats (blue squares, n = 2) measured by ABR.
(TIF)

Figure S3 **Comparison of ABR in homologous rats from the three strains at 18 days old.** Among the three strains, the ABR (mean ± SD) was highest for AGH-*Ednrb^sl/sl^* rats (white squares) at 8 kHz–32 kHz, followed by LEH-*Ednrb^sl/sl^* (red squares) and F344- *Ednrb^sl/sl^* (green squares) rats.
(TIF)

Figure S4 **PCR genotyping of** ***+/+*** **,** ***sl/+*** **,** ***sl/sl*** **rats in the three strains.** Wild type rats show one band of 511 bp. Heterozygous rats show two bands of 210 bp and 511 bp. Homologous *sl* rats show a single band of 210 bp.
(TIF)

Table S1 **Thirty-three microsatellite markers used to examine the congenic extent in chromosome 15.**
(TIF)

Table S2 **Genotypic distribution in six different populations.** Using χ^2^ examination, these populations were confirmed to follow the Mendelian rule.
(TIF)

## References

[1] Skinner MA (1996). Hirschsprung's disease.. Curr Probl Surg.

[2] Amiel J, Sproat-Emison E, Garcia-Barcelo M, Lantieri F, Burzynski G (2008). Hirschsprung disease, associated syndromes and genetics: a review.. J Med Genet.

[3] Passarge E (2002). Dissecting Hirschsprung disease.. Nat Genet.

[4] Heanue TA, Pachnis V (2007). Enteric nervous system development and Hirschsprung's disease: advances in genetic and stem cell studies.. Nat Rev Neurosci.

[5] Walters LC, Cantrell VA, Weller KP, Mosher JT, Southard-Smith EM (2010). Genetic background impacts developmental potential of enteric neural crest-derived progenitors in the Sox10(Dom) model of Hirschsprung disease.. Human Molecular Genetics.

[6] Angrist M, Kauffman E, Slaugenhaupt SA, Matise TC, Puffenberger EG (1993). A gene for Hirschsprung disease (megacolon) in the pericentromeric region of human chromosome 10.. Nat Genet.

[7] Luo Y, Ceccherini I, Pasini B, Matera I, Bicocchi MP (1993). Close linkage with the RET protooncogene and boundaries of deletion mutations in autosomal dominant Hirschsprung disease.. Hum Mol Genet.

[8] Lyonnet S, Bolino A, Pelet A, Abel L, Nihoul-Fekete C (1993). A gene for Hirschsprung disease maps to the proximal long arm of chromosome 10.. Nat Genet.

[9] Amiel J, Attie T, Jan D, Pelet A, Edery P (1996). Heterozygous endothelin receptor B (EDNRB) mutations in isolated Hirschsprung disease.. Human Molecular Genetics.

[10] Attie T, Till M, Pelet A, Amiel J, Edery P (1995). Mutation of the Endothelin-Receptor-B Gene in Waardenburg-Hirschsprung-Disease.. Human Molecular Genetics.

[11] Syrris P, Carter ND, Patton MA (1999). Novel nonsense mutation of the endothelin-B receptor gene in a family with Waardenburg-Hirschsprung disease.. American Journal of Medical Genetics.

[12] Boardman JP, Syrris P, Holder SE, Robertson NJ, Carter N (2001). A novel mutation in the endothelin B receptor gene in a patient with Shah-Waardenburg syndrome and Down syndrome.. Journal of Medical Genetics.

[13] Kusafuka T, Wang YP, Puri P (1996). Novel mutations of the endothelin-B receptor gene in isolated patients with Hirschsprung's disease.. Human Molecular Genetics.

[14] Puffenberger EG, Hosoda K, Washington SS, Nakao K, deWit D (1994). A missense mutation of the endothelin-B receptor gene in multigenic Hirschsprung's disease.. Cell.

[15] Auricchio A, Casari G, Staiano A, Ballabio A (1996). Endothelin-B receptor mutations in patients with isolated Hirschsprung disease from a non-inbred population.. Hum Mol Genet.

[16] Tanaka H, Moroi K, Iwai J, Takahashi H, Ohnuma N (1998). Novel mutations of the endothelin B receptor gene in patients with Hirschsprung's disease and their characterization.. Journal of Biological Chemistry.

[17] Edery P, Attie T, Amiel J, Pelet A, Eng C (1996). Mutation of the endothelin-3 gene in the Waardenburg-Hirschsprung disease (Shah-Waardenburg syndrome).. Nature Genetics.

[18] Hofstra RMW, Osinga J, TanSindhunata G, Wu Y, Kamsteeg EJ (1996). A homozygous mutation in the endothelin-3 gene associated with a combined Waardenburg type 2 and Hirschsprung phenotype (Shah-Waardenburg syndrome).. Nature Genetics.

[19] Angrist M, Bolk S, Halushka M, Lapchak PA, Chakravarti A (1996). Germline mutations in glial cell line-derived neurotrophic factor (GDNF) and RET in a hirschsprung disease patient.. Nature Genetics.

[20] Ivanchuk SM, Myers SM, Eng C, Mulligan LM (1996). De novo mutation of GDNF, ligand for the RET/GDNFR-alpha receptor complex, in Hirschsprung disease.. Human Molecular Genetics.

[21] Salomon R, Attie T, Pelet A, Bidaud C, Eng C (1996). Germline mutations of the RET ligand GDNF are not sufficient to cause Hirschsprung disease.. Nat Genet.

[22] Pingault V, Bondurand N, Kuhlbrodt K, Goerich DE, Prehu MO (1998). SOX10 mutations in patients with Waardenburg-Hirschsprung disease.. Nat Genet.

[23] Southard-Smith EM, Angrist M, Ellison JS, Agarwala R, Baxevanis AD (1999). The Sox10(Dom) mouse: modeling the genetic variation of Waardenburg-Shah (WS4) syndrome.. Genome Res.

[24] Badner JA, Sieber WK, Garver KL, Chakravarti A (1990). A genetic study of Hirschsprung disease.. Am J Hum Genet.

[25] Cohen IT, Gadd MA (1982). Hirschsprung's disease in a kindred: a possible clue to the genetics of the disease.. J Pediatr Surg.

[26] Schuchardt A, D'Agati V, Larsson-Blomberg L, Costantini F, Pachnis V (1994). Defects in the kidney and enteric nervous system of mice lacking the tyrosine kinase receptor Ret.. Nature.

[27] Moore MW, Klein RD, Farinas I, Sauer H, Armanini M (1996). Renal and neuronal abnormalities in mice lacking GDNF.. Nature.

[28] Southard-Smith EM, Kos L, Pavan WJ (1998). Sox10 mutation disrupts neural crest development in Dom Hirschsprung mouse model.. Nat Genet.

[29] Hosoda K, Hammer RE, Richardson JA, Baynash AG, Cheung JC (1994). Targeted and Natural (Piebald-Lethal) Mutations of Endothelin-B Receptor Gene Produce Megacolon Associated with Spotted Coat Color in Mice.. Cell.

[30] Lee HO, Levorse JM, Shin MK (2003). The endothelin receptor-B is required for the migration of neural crest-derived melanocyte and enteric neuron precursors.. Developmental Biology.

[31] Baynash AG, Hosoda K, Giaid A, Richardson JA, Emoto N (1994). Interaction of Endothelin-3 with Endothelin-B Receptor Is Essential for Development of Epidermal Melanocytes and Enteric Neurons.. Cell.

[32] Gariepy CE, Cass DT, Yanagisawa M (1996). Null mutation of endothelin receptor type B gene in spotting lethal rats causes aganglionic megacolon and white coat color.. Proceedings of the National Academy of Sciences of the United States of America.

[33] Karaki H, Mitsui-Saito M, Takimoto M, Oda K, Okada T (1996). Lack of endothelin ETB receptor binding and function in the rat with a mutant ETB receptor gene.. Biochem Biophys Res Commun.

[34] Pingault V, Ente D, Dastot-Le Moal F, Goossens M, Marlin S (2010). Review and update of mutations causing Waardenburg syndrome.. Hum Mutat.

[35] Enomoto H, Araki T, Jackman A, Heuckeroth RO, Snider WD (1998). GFR alpha1-deficient mice have deficits in the enteric nervous system and kidneys.. Neuron.

[36] Matsushima Y, Shinkai Y, Kobayashi Y, Sakamoto M, Kunieda T (2002). A mouse model of Waardenburg syndrome type 4 with a new spontaneous mutation of the endothelin-B receptor gene.. Mammalian Genome.

[37] Ohgami N, Ida-Eto M, Shimotake T, Sakashita N, Sone M (2010). c-Ret-mediated hearing loss in mice with Hirschsprung disease.. Proceedings of the National Academy of Sciences of the United States of America.

[38] Kuriyama S, Mayor R (2008). Molecular analysis of neural crest migration.. Philos Trans R Soc Lond B Biol Sci.

[39] Kenny SE, Tam PKH, Garcia-Barcelo M (2010). Hirschsprung's disease.. Seminars in Pediatric Surgery.

[40] Brooks AS, Oostra BA, Hofstra RM (2005). Studying the genetics of Hirschsprung's disease: unraveling an oligogenic disorder.. Clin Genet.

[41] Newgreen D, Young HM (2002). Enteric nervous system: development and developmental disturbances–part 2.. Pediatr Dev Pathol.

[42] Young HM (2008). Functional development of the enteric nervous system–from migration to motility.. Neurogastroenterol Motil.

[43] McCallion AS, Stames E, Conlon RA, Chakravarti A (2003). Phenotype variation in two-locus mouse models of Hirschsprung disease: Tissue-specific interaction between Ret and Ednrb.. Proceedings of the National Academy of Sciences of the United States of America.

[44] Stanchina L, Baral V, Robert F, Pingault V, Lemort N (2006). Interactions between Sox10, Edn3 and Ednrb during enteric nervous system and melanocyte development.. Developmental Biology.

[45] Shin MK, Levorse JM, Ingram RS, Tilghman SM (1999). The temporal requirement for endothelin receptor-B signalling during neural crest development.. Nature.

[46] Metallinos DL, Bowling AT, Rine J (1998). A missense mutation in the endothelin-B receptor gene is associated with lethal white foal syndrome: an equine version of Hirschsprung disease.. Mammalian Genome.

[47] Yang GC, Croaker D, Zhang AL, Manglick P, Cartmill T (1998). A dinucleotide mutation in the endothelin-B receptor gene is associated with lethal white foal syndrome (LWFS); a horse variant of Hirschsprung disease (HSCR).. Human Molecular Genetics.

[48] Druckenbrod NR, Powers PA, Bartley CR, Walker JW, Epstein ML (2008). Targeting of endothelin receptor-B to the neural crest.. Genesis.

[49] Price ER, Fisher DE (2001). Sensorineural deafness and pigmentation genes: melanocytes and the Mitf transcriptional network.. Neuron.

[50] Ando M, Takeuchi S (1999). Immunological identification of an inward rectifier K+ channel (Kir4.1) in the intermediate cell (melanocyte) of the cochlear stria vascularis of gerbils and rats.. Cell Tissue Res.

[51] Uesaka T, Nagashimada M, Yonemura S, Enomoto H (2008). Diminished Ret expression compromises neuronal survival in the colon and causes intestinal aganglionosis in mice.. J Clin Invest.

