# Correction: Genetic Background Strongly Modifies the Severity of Symptoms of Hirschsprung Disease, but Not Hearing Loss in Rats Carrying *Ednrb^sl^* Mutations

**DOI:** 10.1371/annotation/2ffb2981-5f3f-45b5-a6e1-ae32d92b4993

**Published:** 2011-10-10

**Authors:** Ruihua Dang, Daisuke Torigoe, Sari Suzuki, Yoshiaki Kikkawa, Kanako Moritoh, Nobuya Sasaki, Takashi Agui

The first affiliation should be: Laboratory of Laboratory Animal Science and Medicine, Department of Disease Control, Graduate School of Veterinary Medicine, Hokkaido University, Hokkaido, Japan 

