# Supplementary figures and images for: Genetic Background Strongly Modifies the Severity of Symptoms of Hirschsprung Disease, but Not Hearing Loss in Rats Carrying Ednrbsl Mutations
Source: PLoS One. 2011 Sep 7;6(9):e24086. doi: 10.1371/journal.pone.0024086 (PMC3168492; doi:10.1371/journal.pone.0024086)

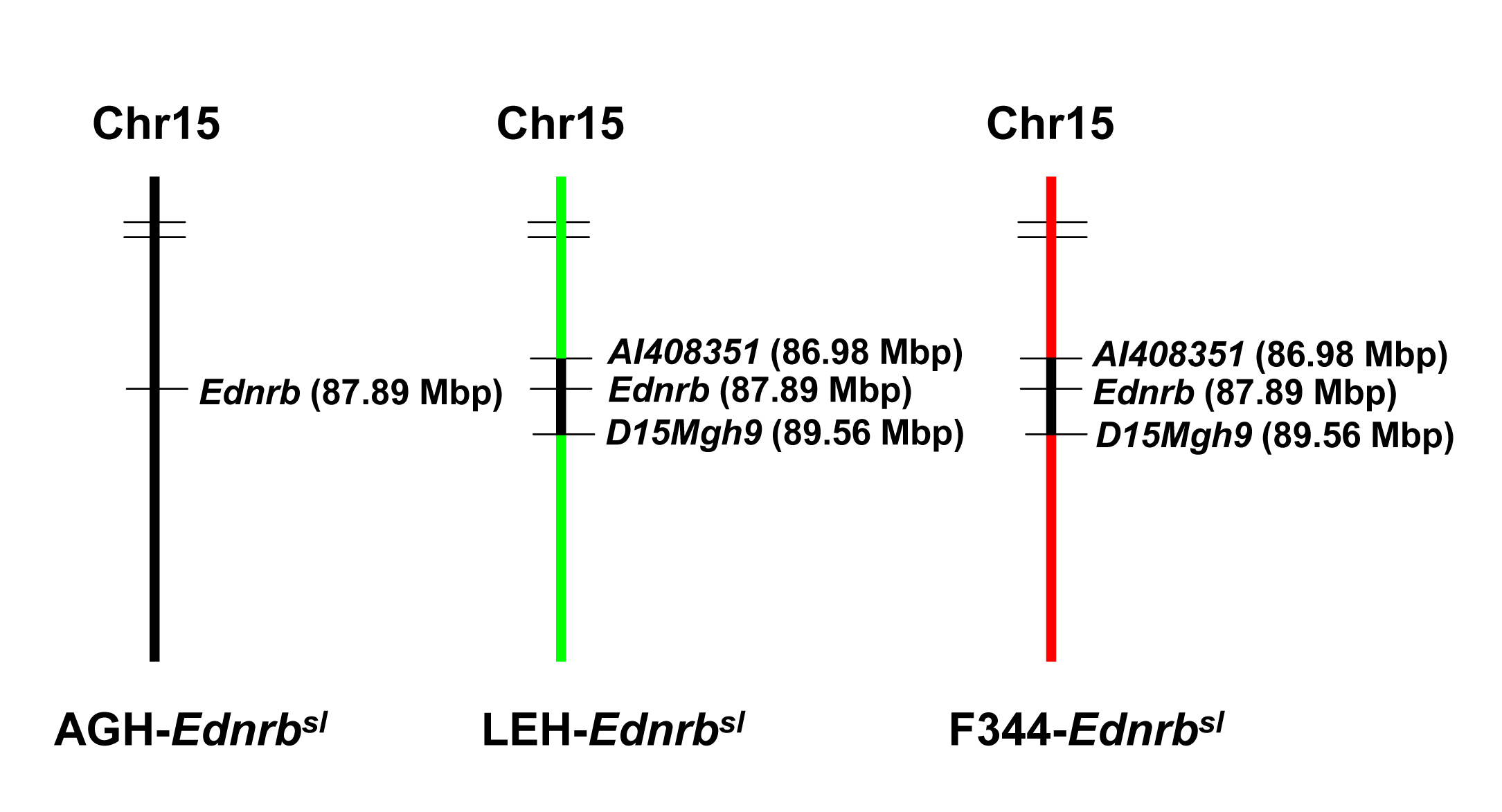

Supplement: Figure S1 — Diagram of LEH- Ednrbsl and F344- Ednrbsl congenic extent. Thirty-three microsatellite markers located on chromosome 15 in the rat were used to examine the congenic extent of LEH-Ednrbsl and F344-Ednrbsl rats. Genetic background in both strains of rat was replaced by the 3 cM region around the Ednrb gene. (TIF) [file pone.0024086.s001.tif]

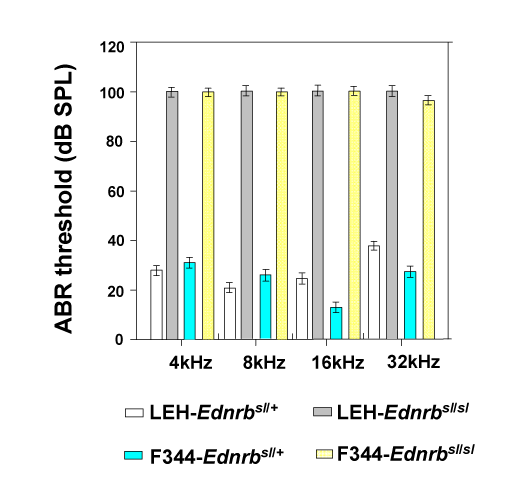

Supplement: Figure S2 — Hearing levels (mean ± SD) in 10-week-old LEH-Ednrbsl/sl rats (gray squares, n = 2) and littermate heterozygous rats (white squares, n = 4), as well as in F344-Ednrbsl/sl rats (yellow squares, n = 4) and littermate heterozygous rats (blue squares, n = 2) measured by ABR. (TIF) [file pone.0024086.s002.tif]

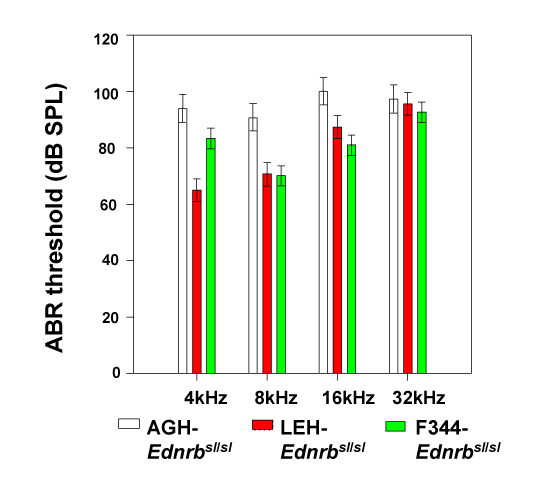

Supplement: Figure S3 — Comparison of ABR in homologous rats from the three strains at 18 days old. Among the three strains, the ABR (mean ± SD) was highest for AGH-Ednrbsl/sl rats (white squares) at 8 kHz–32 kHz, followed by LEH-Ednrbsl/sl (red squares) and F344- Ednrbsl/sl (green squares) rats. (TIF) [file pone.0024086.s003.tif]

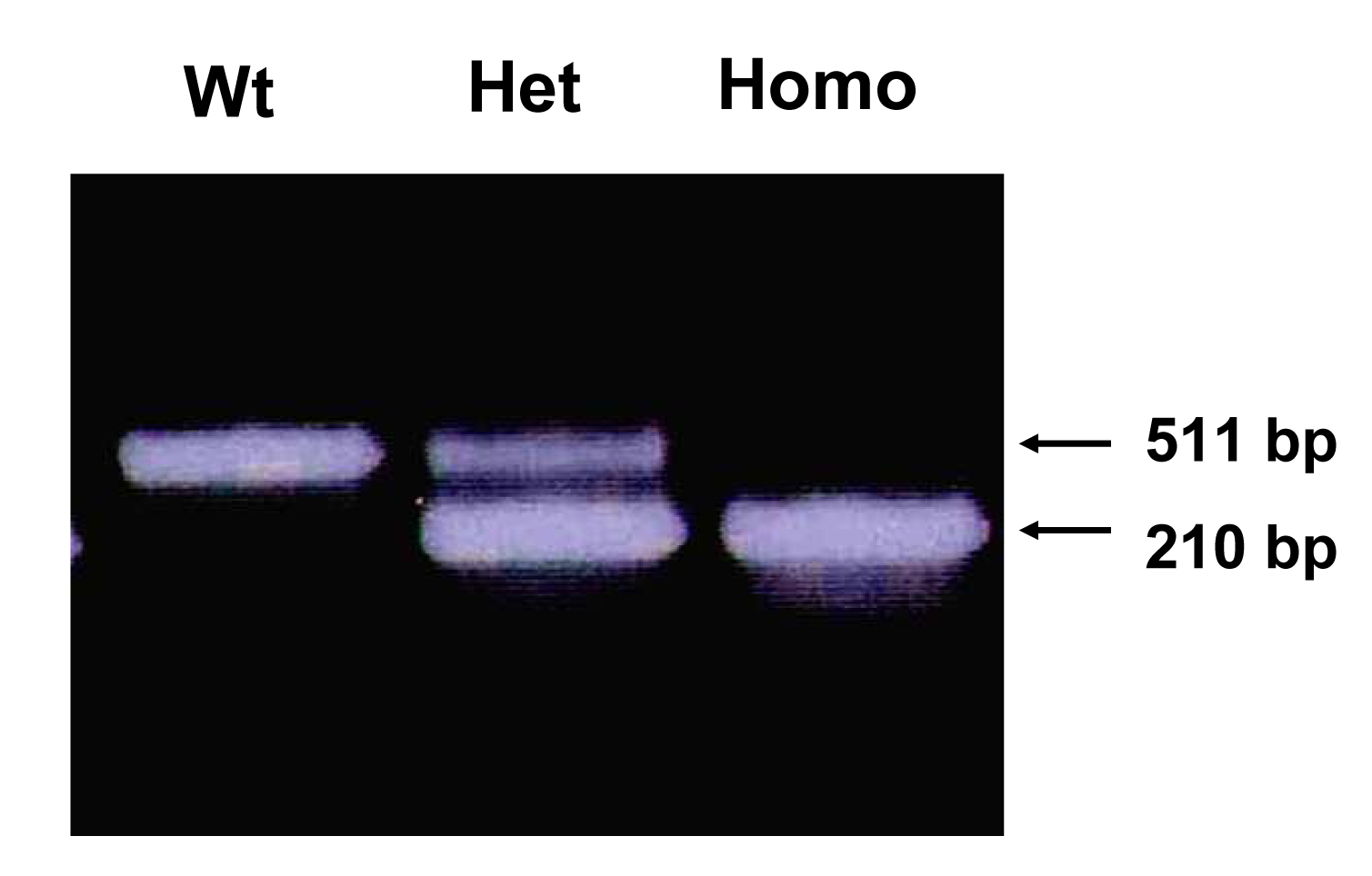

Supplement: Figure S4 — PCR genotyping of +/+ , sl/+ , sl/sl rats in the three strains. Wild type rats show one band of 511 bp. Heterozygous rats show two bands of 210 bp and 511 bp. Homologous sl rats show a single band of 210 bp. (TIF) [file pone.0024086.s004.tif]

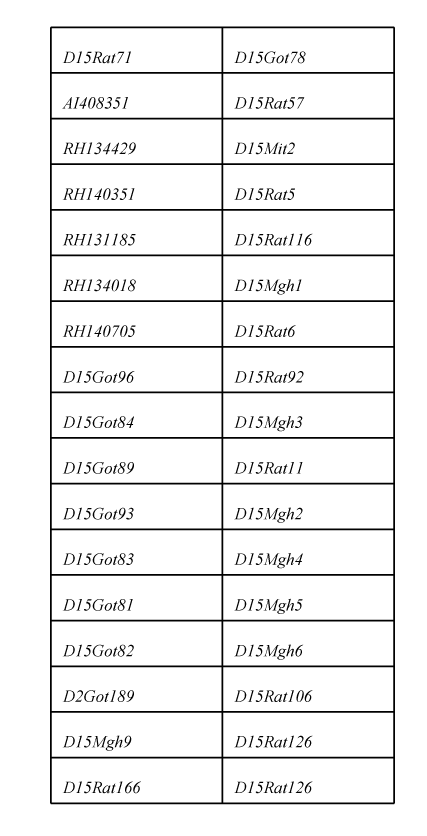

Supplement: Table S1 — Thirty-three microsatellite markers used to examine the congenic extent in chromosome 15. (TIF) [file pone.0024086.s005.tif]

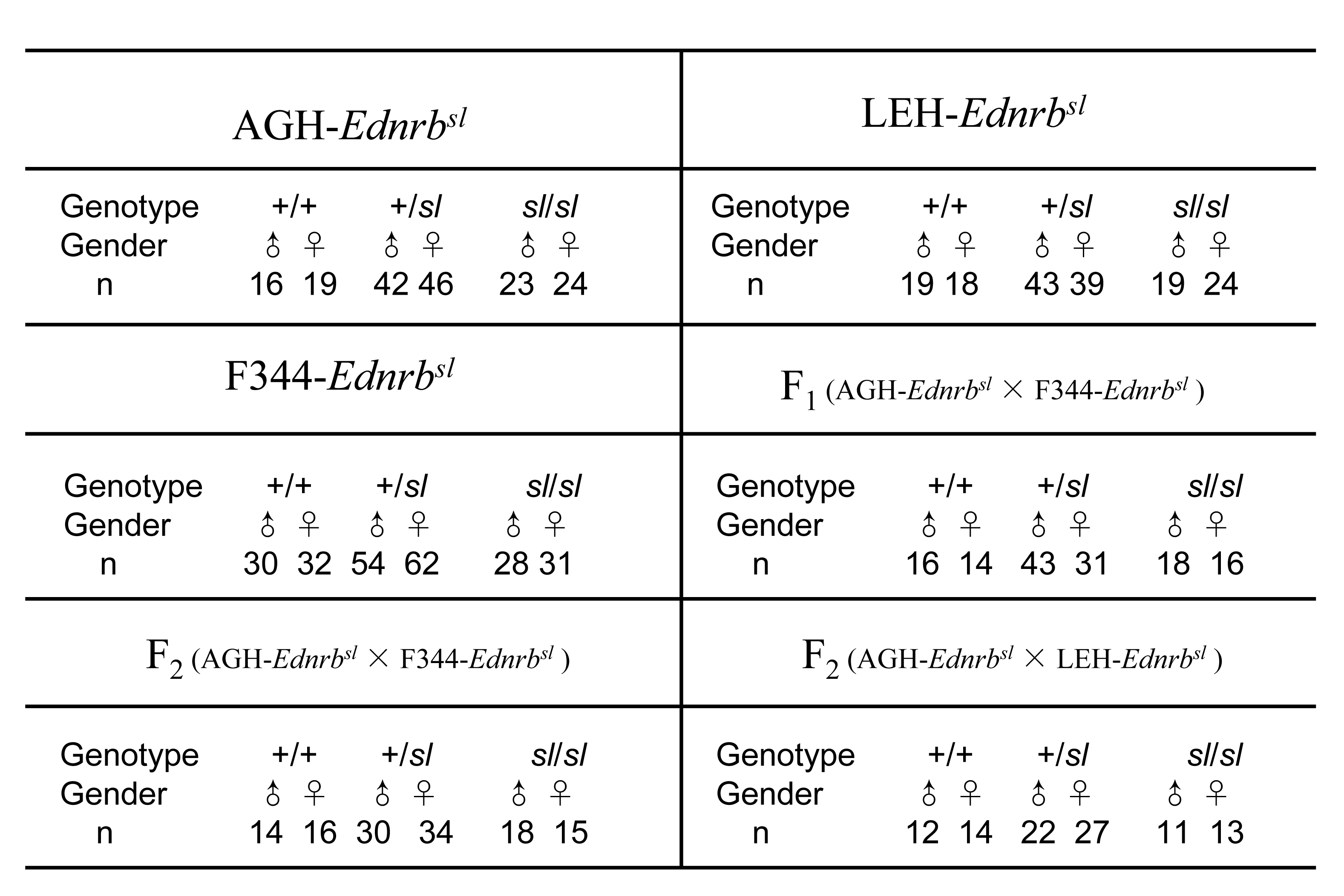

Supplement: Table S2 — Genotypic distribution in six different populations. Using χ2 examination, these populations were confirmed to follow the Mendelian rule. (TIF) [file pone.0024086.s006.tif]
